# Wealth Status, Health Insurance, and Maternal Health Care Utilization in Africa: Evidence from Gabon

**DOI:** 10.1155/2020/4036830

**Published:** 2020-01-11

**Authors:** N'doh Ashken Sanogo, Sanni Yaya

**Affiliations:** University of Parakou, Parkou, Benin

## Abstract

**Background:**

To achieve the universal health coverage among other Sustainable Development Goals, African countries have shown the commitment by implementing strategies to improve access and coverage of health care services whose access is still very low. The achievement of universal health care requires the provision and availability of an adequate financing system. This study explored the wealth-related association of compulsory health insurance on maternal health care utilization in Gabon.

**Methods:**

The study used the 6^th^ round of Gabon Demographic and Health Surveys (GDHSs)—2012 data to explore three outcome measures of maternal health care utilization extracted on number of antenatal care (ANC) visits during pregnancy, place of birth delivery, and postnatal health care. The dependent variable was women with health insurance coverage against those without. Logistic regression and propensity scoring matching analysed associations of health insurance coverage on women's utilization of health care.

**Results:**

Mean (+/− SD) age of women respondents of reproductive age was 29 years (9.9). The proportion of at least 4 antenatal care visits was 69.2%, facility-based delivery was 84.7%, and postnatal care utilization was 67.9%. The analysis of data showed disparities in maternal health care services utilization. The GDHS showed maternal age, and geographical region was significantly associated with maternal health care service utilization. A high proportion of urban dwellers and Christian women used maternal health care services. According to the wealth index, maternal health services utilization was higher in women from wealthy households compared to lower households wealth index (ANC (Conc. Index = 0.117; *p* ≤ 0.001), facility-based delivery (Conc. Index = 0.069; *p* ≤ 0.001), and postnatal care (Conc. Index = 0.075; *p* ≤ 0.001), respectively). With regard to health care insurance coverage, women with health insurance were more likely to use ANC and facility-based delivery services than those without (concentration indices for ANC and facility-based delivery were statistically significant; ANC: *z*-stat = 2.69; *p*=0.007; Conc. Index: 0.125 vs. 0.096 and facility-based delivery: *z*-stat = 3.38; *p*=0.001; Conc. Index: 0.076 vs. 0.053, respectively).

**Conclusion:**

Women enrollment in health insurance and improved household's financial status can improve key maternal health services utilization.

## 1. Background

In the attempt to meet Millennium Development Goals (MDGs) by 2015, Africa witnessed improvements in the overall health of its population over the past 20 years [1]. Life expectancy at birth rose from 50 years in 1990 to 60 years by 2015. Adults' mortality rate decreased from 361 to 300 deaths per 100 000 people, which was a reduction of 61 deaths per 100 000 people. The under-5 mortality rate and the maternal mortality rate declined by 54.2% and 40.7%, respectively [1, 2]. Regarding infectious diseases, HIV prevalence declined by 57%, malaria incidence declined by 42%, and mortality rates from tuberculosis declined by 31% [1, 2].

Reproductive health and maternal outcomes are one of the areas where strides were made, with declines in maternal mortality and morbidity across the continent [1, 2]. However, despite improvements in maternal health across Africa, such improvements vary markedly between and within regions of the continent and countries. There are still various inequities and disparities in access to adequate care, such as maternal care, with research evidence indicating the uneven distribution of initiatives, programs, and their effects as a major determinant [1, 2]. According to the World Health Organization (WHO), about 400 million people globally do not have access to basic quality health services, and 6% of the people living in low- and middle-income countries experience extreme poverty as a result of payment for health services [3]. In Africa, accessibility and coverage of essential health services are therefore low. For instance, only 43% of pregnant women have the four recommended prenatal visits compared to the global average of 55% [3, 4]. Only 49% of births are attended by skilled health personnel compared to the global average of 70% [3, 4]. In sub-Saharan Africa, the rate of postnatal care is 53% compared to 86% for North Africa [5]. Direct payments, which refer to fees levied for consultations with health professionals, are a major cause of this situation across the continent [1]. For example, studies have shown that direct payments for care provide limited access to care for the poor and women [6–8]. The continent has been undergoing transitions in various areas, such as demography, economy, and societal makeup that have resulted in new health expectations [1, 2]. This is compounded by health threats whose adverse effects on African populations are magnified due to feasible movement across the continent. For increased budgetary allocation and initiatives to have successful effects on health, including maternal health, improvements need to be made in health services provision to the public [1].

For a sustainable solution, WHO members agreed to target Universal Health Coverage (UHC) for their populations [9]. UHC is defined as ensuring that all people can use the needed promotive, preventive, curative, rehabilitative, and palliative health services of adequate quality to be effective, while also ensuring that the use of these services does not expose the user to financial hardship [1, 2, 9]. Central to the health targets of the new Sustainable Development Goals (SDGs), achieving UHC (SDG target 3.8) is a major priority for African countries [1]. To achieve UHC and improve access to care, the WHO recommends prepayment of health care in which direct payments and user fees are drastically reduced [9–11]. The organization further recommends a broad and equitable tax system, compulsory health insurance, or both [10, 11].

In line with this goal, some countries in the continent are implementing strategies to improve access to and coverage of health services. Many other countries have made commitments to take measures towards achieving UHC [3]. Gabon is a member country of WHO that committed in 2005 to achieve UHC. It is in this context that the country adopted a political reform to reach UHC by the creation of the NFHISG (National Fund for Health Insurance and Social Guarantee of Gabon) in 2008 [12–15]. The NFHISG aims to provide health coverage for the Gabonese populations, especially those who are vulnerable, poor, and living in poverty, by reducing health costs and pooling financial resources [16]. Health care services are provided by public and private care providers of the primary, secondary, and tertiary levels for outpatient care and hospitalizations. For health care provided through public, private, or nonprofit channels, the NFHISG is essentially the health financing instrument and the risk pooling instrument as it spreads and shares financial risks across a population to provide financial risk protection for each individual seeking care [17]. The NFHISG has enabled the health system to improve the supply and quality of care, such as antenatal care, by recruiting more staff and remunerating equipment and reagents [16]. Membership in this compulsory health insurance scheme was progressive, starting with the indigent (households earning less than 80,000 XAF per month or US $ 146) in 2008, public sector workers in 2010, and finally, those of the private and parapublic sector in 2011 [13, 14]. At the end of 2012, the indigent coverage rate was 79%. In 2015, Gabon had a maternal mortality ratio of 291, which has improved from previous years [18]. However, the proportion of maternal deaths among deaths of female reproductive age increased in 2015 (8.6%) from the proportions in 2005 (7.8%) and 2010 (7.4%). Today, 78% of women make at least 4 antenatal care visits during pregnancy [17].

In this context, with compulsory health insurance on rise on the continent and for performant health systems, access to quality health care is essential. A number of rigorous studies have evaluated the impact of health insurance on the use of general health care, but there is limited empirical evidence of the impact of compulsory health insurance on use of maternal health care [12–14]. A study of the impact of health insurance on maternal health care utilization in Ghana, Indonesia, and Rwanda shows positive effects of health insurance coverage on maternal health care utilization [15]. Health insurance coverage contributed to an 8% point increase in access to four or more antenatal care visits in Ghana and a 3% point increase in Indonesia, as well as a 5–11 percentage point increase in use of facility-based delivery care in all three countries [15]. Another study of the impact of health insurance in maternal care utilization in Ghana shows that, after adjusting for socioeconomic, demographic, and obstetric factors, the likelihood among insured women of having antenatal care increased by 96% and of skilled delivery by 129%, while postnatal care increased by 61% [16]. In Bangladesh, a free maternal health care program shows that women in intervention areas had significantly higher probability of antenatal care utilization, facility-based delivery, and postnatal care [17].

In Gabon, since the introduction of compulsory health insurance ten years ago, no study has been done to assess the use of care provided to the populations covered [13, 14]. For this reason, there is a growing need to evaluate whether health insurance has contributed to greater use of maternal health care [13, 14]. For instance, maternal health is an important nonincome indicator of poverty, which means that reducing poverty in developing countries is to improve the health of women [18]. Wealth means the economic status of the individual or household [18]. A Ghanaian study shows a positive relationship between professional antenatal care coverage and wealth quintile, with women in the highest wealth quintile more likely to receive care from a health professional than those in the lowest wealth quintile [19]. Another study in Turkey shows household wealth is positively and significantly associated with choosing health facility for delivery [12]. A Malian study explains that household poverty and personal problems to be negatively related to the use of maternal health care [20]. In Sierra Leone, a study examining the impact of the free health care initiative shows that free health care improves access to and utilization of maternal and child health services but is insufficient in addressing wealth-related inequity that exists for institutional deliveries [21]. Using nationally representative data from the Demographic and Health Surveys (DHSs), this paper assesses the wealth-related modifying effect of the Gabonese compulsory health insurance on use of antenatal care, delivery in health care institution, and postnatal care by Gabonese women.

## 2. Methods

### 2.1. Study Settings

The research setting for this study was Gabon, a Central African country that straddles the equator. It has a population of 1, 811 079 million, of which 87% reside in urban areas [12]. It is a relatively young population with an average age of 26 and about half the population younger than 22 years of age. The country has high education enrollment and literacy rates, and Christianity is the predominantly practiced religion amongst its citizens [12]. It is considered an upper middle-income country, but human development indicators and outcomes are similar to countries with a lower income. Approximately one-third of the population is living in poverty [12].

### 2.2. Data Extraction

This study utilized data from the 6^th^ round of the Republic of Gabon's Demographic and Health Surveys (DHSs), 2012. A total of 8,422 reproductive aged women were interviewed in the survey. In more than 90 developing countries, DHSs have been technically assisting the implementation of over 300 surveys, which have provided nationally representative data as well as comparative data on health and population. There has been deficiency on pragmatic data on the consequence of health insurance on women health services coverage in many Gabon's regions. These data collected from the respondents who were aged 15–49 years in the survey were used to describe levels of utilization of health care services and also their coverage for health insurance. Information on the women sexual behaviours and outcomes of their sexual and reproductive practices, their socioeconomic status, as well as the household characteristics were also obtained. Women who had live birth in the preceding five years to the survey were the target for the assessment of the consequence of health insurance. For sampling procedure of the DHS, refer the 6^th^ round of Gabon Demographic and Health Surveys (GDHSs)—2012 Final Report.

## 3. Variables Selection and Measurement

### 3.1. Outcome Variables

Three outcome variables for maternal health care utilization were explored in this study. They are as follows: (1) number of visits to antenatal care throughout the period of pregnancy. We categorized this dichotomously as “adequate” when at least four ANC visits were made and “inadequate” when less than four ANC visits were made. (2) Deliveries that took place at home vs those that took place at the health facilities. We classified this as binary outcome 0 if the delivery took place at home and 1 if it took place at health facilities. (3) The postnatal care services of the respondents were measured using the postnatal care attendance. According to the WHO, postnatal care is defined as care received by women and newborns during the first six weeks after birth [4].

### 3.2. Treatment Dependent Variable

This was measured for the health insurance coverage women category vs. the none health insurance coverage category women.

### 3.3. Explanatory Variables

According to literature search and availability of the datasets, the following covariates were included in the analysis: age groups (15–24, 25–29, 30–34, 35–39, 40–44, and 45–49), residency (urban and rural), region (Libreville-Port-Gentil, Estuaire, Haut-Ogooue, Moyen-Ogooue, Ngounie, Nyanga, Ogooue Maritime, Ogooue-Ivindo, Ogooue-Lolo, and Woleu-Ntem), religion (Christianity, Islam, other religions, and no religion), sex of the household head (male/female), educational status (no education, primary, secondary, and higher), wealth status (poorest, poorer, middle, richer, and richest), and occupation (unemployed and employed). The analysis included also media use such as newspaper/magazine (yes/no), radio use (yes/no), and TV use (yes/no). We included also marital status (never married and married/living with a partner), wanted last child (then, later, and no more), age at first birth (<18 years, 18–25 years, and ≥26 years), parity (nil, 1–3, and ≥4), women's decision-making power (low, moderate, and high), and health insurance coverage (yes/no). The wealth quintile was a measure of the overall household income which was categorized into quintiles that classify the households into five groups from poorest to richest (quintile 1/poorest, quintile 2/poorer, quintile 3/middle, quintile 4/richer, and quintile 5/richest).

### 3.4. Ethical Considerations

The study required no ethical approval from any institution since we used DHS data which are publicly available. The institutions that funded, commissioned, and manage the database have the responsibility for ethical issues. More so, the ICF International and Institutional Review Board (IRB) approved all DHS surveys in all the countries.

### 3.5. Statistical Analysis

Percentage and means (±SD) were used to summarize and examine the sociodemographic distributions and maternal economic characteristics. Complex survey module (svyset) was used for data representation adjustment to account for clustering, stratification, and sample weight for all the analyses. Outcome variables in percentages were represented in bar charts. Logistic regression and propensity scoring matching (PSM) were used in the evaluation of the consequence of the coverage of health insurance on women health care utilization. The propensity to sought health care services was correlated as well as factors which influenced the tendency to enroll in health insurance, and these may have introduced a sort of prejudice due to observed and unobserved heterogeneity. The strength of PSM is that the selection bias is not addressed by the regression model. Therefore, we used a binary exposure variable (coverage by compulsory health insurance: yes vs no), a set of potentially confounding covariates (treatment independent variables) that are determinants of both exposure and response were adjusted for. We used the concentration index and the Lorenz curves to determine the association between health insurance coverage and utilization of maternal health services. Details on how this is determined and interpreted have been published elsewhere [22, 23]. Data were analysed at 5% significance level using the 14^th^ version of Stata (StataCorp, College Station, TX).

## 4. Results

The mean (SD) age of respondents for women of reproductive age was 29 years (9.9). The number of women reported in the survey decreased by increase in age. About two-thirds (68.1%) of the respondents were urban dwellers, while majority of them were of Christianity religious belief (86.4%). Libreville-Port-Gentil region accounted for the highest proportion of respondents (18.5%), and this was followed by Ogooue-Ivindo (11.7%), Haut-Ogooue, Ngounie (10.3%), and Estuaire (10.0%), respectively. Almost 60% of the women came from economically disadvantaged households. Majority of the women were unemployed (58.0%) and from households with male headship (63.7%). Approximately 5% of respondents had no formal education and had higher education, respectively. Majority of women watch TV (83.3%), and two-thirds of them were currently married/living with a partner; while more than half of respondents (56.4%) wanted last child then and had health insurance coverage (54.2%). One-third of the women had at least 4 children ever born and only about 5% of them were at least aged 26 years at first birth (Table 1).

**Figure 1:**
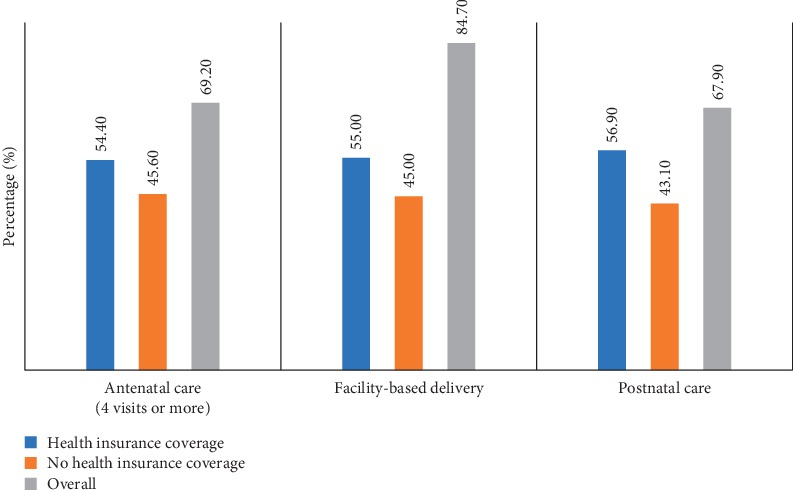
Differences in maternal health care utilization by health insurance coverage.

The differences in maternal health care utilization by health insurance coverage are presented in Figure 1. Antenatal care (4 visits or more), facility-based delivery, and postnatal care utilization were 8.8%, 10%, and 13.8% higher among women in health insurance coverage, compared to those with no health insurance coverage. Overall, ANC (4 visits or more), facility-based delivery, and postnatal care were 69.2%, 84.7%, and 67.9%, respectively, among women of reproductive age.

The results showed disparities in maternal health care utilization across respondents' characteristics such as maternal age and geographical region amongst others. Overall, urban dwellers and women of Christianity beliefs had a higher proportion of maternal health care utilization (about 70% or more). Poor (poorest and poorer) women and the unemployed reported slightly above 50% ANC, facility-based delivery, and postnatal care utilization. Libreville-Port-Gentil gained prominence among all geographical regions in maternal health care utilization. Respondents who reportedly watching TV or those currently married/living with a partner, wanted last child then, and have 1–3 children had improved maternal care service utilization, as shown in Table 2.

Based on the results from Table 3, the unadjusted model showed that women from poorest households, who had health insurance coverage had 21% significant reduction in facility-based delivery, compared to women who had no health insurance coverage (OR = 0.79; CI: 0.63, 0.99). Conversely, women from richest households, who had health insurance coverage, were 2.48 times as likely to adequately utilize antenatal care, compared to their counterparts who had no health insurance coverage (OR = 2.48; CI: 1.06, 5.83), respectively. Furthermore, women from richest households who have health insurance coverage had higher utilization level for facility-based delivery and postnatal care in the average treatment effect in the population model, compared with women from richest households who had no health insurance coverage (facility-based delivery: *β* = 0.05; CI: 0.01, 0.10 and postnatal care: *β* = 0.11; CI: 0.01, 0.20). Similarly, from the average treatment effect on the treated model, women from richest households, who have health insurance coverage had increase in postnatal care, compared with women from richest households who had no health insurance coverage (*β* = 0.18; CI: 0.07, 0.29).

Results from Table 4 showed significant difference in the utilization level of maternal health care services across the household wealth index. The concentration index, which was directly related to Lorenz curves, quantified the degree of wealth-related inequalities in ANC, facility-based delivery, and postnatal care. Overall, maternal health care utilizations were significantly more in the higher household wealth, compared to the lower household wealth groups; ANC (Conc. Index = 0.117; *p* ≤ 0.001), facility-based delivery (Conc. Index = 0.069; *p* ≤ 0.001), and postnatal care (Conc. Index = 0.075; *p* ≤ 0.001), respectively. However, the test for differences between women who are covered by health insurance vs. those not covered by health insurance, concentration indices for ANC, and facility-based delivery were statistically significant; ANC: *z*-stat = 2.69; *p*=0.007; Conc. Index: 0.125 vs. 0.096 and facility-based delivery: *z*-stat = 3.38; *p*=0.001; Conc. Index: 0.076 vs. 0.053, respectively.

**Figure 2:**
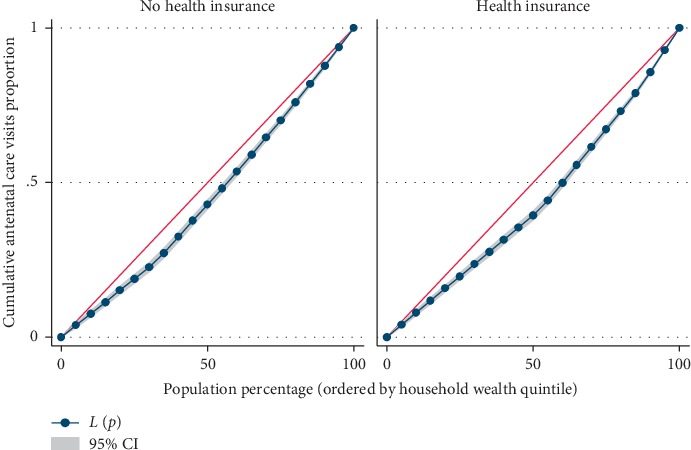
Antenatal care visits utilization by household wealth quintile.

**Figure 3:**
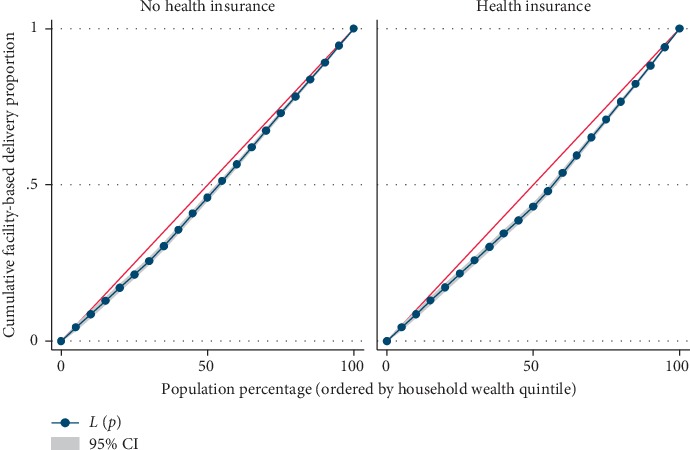
Facility-based delivery utilization by household wealth quintile.

**Figure 4:**
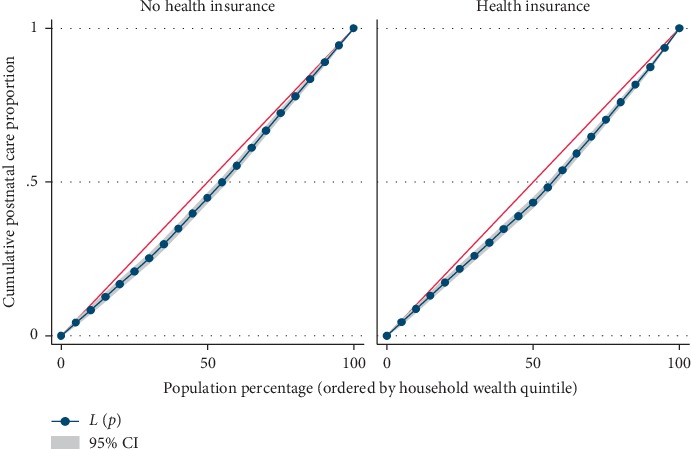
Postnatal care utilization by household wealth quintile.

Figures 2–4 shows the household wealth-related inequalities for the utilization of ANC, facility-based delivery, and postnatal care by health insurance health coverage. All figures indicated that women covered by health insurance with a high household wealth-related group had more maternal health care utilization. Our result also shows that the Lorenz curve is further away from the line equality. The inequalities in household wealth level were more among women with health insurance who utilized adequate ANC, facility-based delivery, and postnatal care, as the areas between the curve and the line of inequality were maximal (Figures 2–4).

## 5. Discussion

This study explored the patterns of ANC utilization and visits, delivery at the health facilities, and postnatal care visits of reproductive age women in Gabon. While approximately two-thirds of women were reported to have postnatal care and at least four ANC visits, the prevalence of delivery at health facility was relatively high. These findings from this study are in agreement with the results of some studies previously conducted in some sub-Saharan Africa countries where utilization of maternal and child health care services were extensively examined [24–26]. Furthermore, the effect health insurance coverage bears on maternal health services was examined both sociodemographically and using wealth indices of the households. Based on the findings, there were disparities in the maternal utilizations of health care services, including visits to ANC, delivery at the health facility, and attending postnatal care among women of reproductive age by socioeconomic factors. The use of maternal health care services was higher among unemployed Christian women in urban areas of Libreville-Port-Gentil. Maternal health care services utilization was also higher when male heads of households and/or pregnant women were educated and married and had access to a TV. The findings are in corroboration with other previous studies where there are need for improvement of maternal health care indicators across notable categories of sociodemographic factors [27–29]. This is consequent upon the fact that women of certain categories of selected characteristics were observed to exhibit an increase in the use of maternal health care services, when compared with women of other categories.

In addition, maternal utilizations of ANC visits, delivery at the health facility, and postnatal care visits were observed to be positively and significantly associated with the wealth index of the household. There was a significant difference in the use of maternal health care and the wealth index of the household using the concentration index model. Universal Health Coverage as a concept majorly targets to support reduction in inequalities in health care utilization of mothers, specifically to get to the most disadvantaged women. Findings from this study are evident that women in the lower household wealth index had reduction in the use of maternal health care services. This brings to limelight that there is disproportionate share in maternal health care by disadvantaged women, irrespective of the high demand in most poor resource settings. Despite several efforts by agencies to intervene in the improvement of maternal health care services in SSA countries including Gabon, the results obtained for ANC visits, facility-based delivery, and postnatal care by household wealth index showed that substantial gap exists between the well-off and disadvantaged. This explains the economic differences in accessing maternal or reproductive care services, though other factors, such as satisfaction in health care services, travel time to health facility, waiting time to receive services, and quality of care are also determining factors in health care utilizations [30]. This presupposes that economic empowerment could enhance the women ability to have or develop a good health care-seeking behaviour. These findings agree with several other studies previously conducted on maternal socioeconomic status such as the wealth index of the households [31–33].

Based on the results from the Lorenz curve, we found a high degree of inequality, compared to a straight diagonal representing perfect equality—it shows the Lorenz curve is further away from the equality line. The inequalities in the household wealth level were more among women with health insurance who utilized adequate ANC, facility-based delivery, and postnatal care, as the areas between the curve and the line of inequality were maximal. The gap in the visit to the ANC clinic for a minimum of four times, delivery at health facility, and attending postnatal care after delivery, between the poor and rich women is wider among people who are in the enrollment of health insurance, compared to those without coverage for health insurance. Health insurance programmes in several resource-constrained settings are doubtless in the initial phases though attentions are increasingly been attracted to help in enhancing access to health care services in these areas, through provision of acceptable, accessible, and affordable health care services for the people [34]. Due to increase in demand for maternal health care among Gabonese women of the reproductive age group, Gabon is already having a widespread maternal health care insurance coverage especially in the area of childbearing. Health insurance premiums are one factor influencing the use of maternal health care services. Some other numerous factors also play some significant role in health care such as cultural factors, apparent need for utilization, knowledge of what health insurance is all about and the benefits accrued to it, and individual's health condition [35, 36]. It is worthy of note that the mechanisms of health insurance scheme in Gabon are such that improvements are made in accessibility of finance for health care specifically for those financially disadvantaged women.

In sum, this shows the importance of the establishment of compulsory health insurance in developing countries such as Gabon. It can serve as evidence for the policies by showing that access to care and protection against financial risk are a major strategy to improve maternal key ANC visits, facility-based delivery, and postnatal care.

## 6. Strengths and Limitations

The greatest strength that this study has is the use of the nationally representative large dataset. This doubtlessly confers on its findings the benefits to be used as a general condition of Gabonese women who are in the reproductive age group. More so, this is one of the prime analyses that explores the nationwide health insurance coverage in relation to visits to antenatal care (ANC) during the period of pregnancy, visiting health facility for delivery, and going for postnatal clinic after delivery. The finding of this study is believed to serve as a standard or yardstick and inducement for additional studies at the national scale on associated subject matters. The foremost weakness or limitations of this study is that the data used are a cross-sectional one, and our inability to measure sources of demand-side unnoticed heterogeneity in the secondary data may have caused bias in the estimates we made as regards correlations of the maternal health care services. Additionally, the unobtainability of some pertinent variables that can make this work robust was also a constraint in DHS data usage. Also, DHS had no report on whether maternal health care services were available and accessible to the mothers and also how frequently they are used by the Gabonese women. To finish, there is a risk of recall bias given that women were asked about events that happened within the 5 years prior to the conduct of the survey. The fact that women who gave birth to their last child in the 5 years before the conduct of the survey were included in the study may have reduced the number of women who are rich and living in urban areas knowing that these groups of women are more likely to give birth to fewer children.

## 7. Conclusion

We have identified the impact of health insurance coverage on maternal health care services and associated household wealth-related factor in Gabon. The findings proved that getting women to enroll in health insurance is a major strategy to improve the utilization of some important maternal health services, which include adequate ANC visits, delivery at health facilities, as well as attending postnatal care clinic. Generally, the findings of this study point to the fact that there is a significant increment in the use of standards recommended for adequate maternal health care which can be attributable to coverage of health insurance. Findings from this study will serve as evidence that can help in the formation of policy for health insurance. This can be done by using some laborious approaches to validate the impression that health insurance and household wealth quintile create on maternal health. Furthermore, improving the household wealth index through economically empowerment of women is vital in the improvement of maternal health care utilization. Health insurance coverage and women's empowerment programme will be helpful to achieve equality in health coverage, irrespective of women's status. In addition, formulating economic health care policies and strengthening health care programmes such as health insurance coverage will help to improve the use of maternal care services and also address the differences in the use of these services. Finally, the government should ensure that women of reproductive age are compulsorily enrolled in health insurance and provided with behaviour change communication using the mass media approach to understand the need, designs, or forms of maternal health care and its benefits.

## Figures and Tables

**Table 1:** Percentage distribution of respondents' characteristics (*n* = 8,422).

Variable	*n*	%
*Age (29* *±* *9.9)*		
15–19	1834	21.8
20–24	1573	18.7
25–29	1294	15.4
30–34	1102	13.1
35–39	1008	12.0
40–44	866	10.3
45–49	745	8.9

*Place of residence*		
Urban	5736	68.1
Rural	2686	31.9

*Region*		
Libreville-Port-Gentil	1557	18.5
Estuaire	844	10.0
Haut-Ogooue	875	10.4
Moyen-Ogooue	681	8.1
Ngounie	871	10.3
Nyanga	672	8.0
Ogooue Maritime	560	6.7
Ogooue-Ivindo	984	11.7
Ogooue-Lolo	741	8.8
Woleu-Ntem	637	7.6

*Religion*		
Christianity	7273	86.4
Islam	373	4.4
Other religions	103	1.2
No religion	665	7.9

*Wealth index*		
Poorest	3076	36.5
Poorer	1906	22.6
Middle	1302	15.5
Richer	1141	13.6
Richest	997	11.8

*Working status*		
Currently employed	3532	42.0
Unemployed	4873	58.0

*Sex of the household head*		
Male	5361	63.7
Female	3061	36.3

*Reading newspaper/magazine*		
Yes	3884	46.2
No	4526	53.8

*Listening to radio*		
Yes	4288	51.0
No	4128	49.0

*Watching TV*		
Yes	7008	83.3
No	1406	16.7

*Level of education*		
None	383	4.6
Primary	2870	34.1
Secondary	4818	57.2
Higher	351	4.2

*Marital status*		
Never married	2765	32.8
Currently married/living with a partner	5657	67.2
*Wanted last child*		
Then	2325	56.4
Later	1508	36.6
No more	292	7.0

*Age at first birth*		
<18 years	3003	47.1
18–25 years	3083	48.3
≥26 years	297	4.6

*Parity*		
Nil	2039	24.2
1–3	3682	43.7
≥4	2701	32.1

*Women's decision-making power*		
Low	677	34.8
Moderate	667	34.2
High	604	31.0

*Health insurance coverage*		
Yes	4550	54.2
No	3844	45.8

**Table 2:** Health care services utilization across maternal characteristics (*n* = 8,422).

Variable	Antenatal care (≥4 visits) (%)	Facility-based delivery (%)	Postnatal care (%)	Health insurance coverage (%)
*Age*				
15–19	12.2	12.9	12.6	17.7
20–24	26.1	25.4	24.8	16.1
25–29	23.4	22.6	22.3	14.8
30–34	17.9	17.6	18.1	14.1
35–39	12.2	12.5	13.3	13.6
40–44	6.5	7.1	6.7	12.7
45–49	1.7	1.9	1.9	11.0

*Place of residence*				
Urban	71.8	70.2	70.1	62.8
Rural	28.2	29.8	29.9	37.2

*Region*				
Libreville-Port-Gentil	18.5	17.6	18.6	11.5
Estuaire	9.9	9.7	9.3	8.4
Haut-Ogooue	13.8	12.2	9.5	13.0
Moyen-Ogooue	5.8	7.9	8.2	8.9
Ngounie	9.9	11.0	12.6	12.6
Nyanga	8.4	8.7	9.5	7.7
Ogooue Maritime	7.7	7.3	8.5	5.3
Ogooue-Ivindo	9.2	9.6	10.5	16.1
Ogooue-Lolo	10.5	9.6	9.2	10.9
Woleu-Ntem	6.4	6.4	4.3	5.9

*Religion*				
Christianity	84.7	85.4	84.8	88.3
Islam	6.3	5.8	6.2	1.6
Other religions	1.3	1.1	1.3	1.1
No religion	7.7	7.7	7.7	9.0

*Wealth index*				
Poorest	33.6	36.8	36.7	44.6
Poorer	24.5	24.4	23.7	21.0
Middle	16.6	16.4	16.7	12.9
Richer	13.9	12.4	12.6	10.6
Richest	11.5	10.0	10.3	10.9

*Working status*				
Currently employed	41.0	40.9	42.5	45.6
Unemployed	59.0	59.1	57.5	54.4

*Sex of the household head*				
Male	66.9	66.6	66.8	62.8
Female	33.1	33.4	33.2	37.2

*Reading newspaper/magazine*				
Yes	45.6	45.4	47.1	44.9
No	54.4	54.6	52.9	55.1

*Listening to radio*				
Yes	50.6	50.3	52.3	50.9
No	49.4	49.7	47.7	49.1

*Watching TV*				
Yes	86.9	85.5	86.1	80.0
No	13.1	14.5	13.9	20.0

*Level of education*				
None	4.7	4.6	5.0	3.3
Primary	31.3	32.7	33.7	39.2
Secondary	59.6	58.9	57.2	52.8
Higher	4.4	3.8	4.1	4.8

*Marital status*				
Never married	22.0	22.2	21.7	29.0
Currently married/living with a partner	78.0	77.8	78.3	71.0

*Wanted last child*				
Then	57.2	56.2	58.3	54.6
Later	36.7	36.9	35.0	36.3
No more	6.1	6.9	6.7	9.1

*Age at first birth*				
<18 years	42.4	44.0	43.7	47.4
18–25 years	52.9	51.3	51.3	48.3
≥26 years	4.7	4.7	5.0	4.3

*Parity*				
Nil				19.3
1–3	63.2	61.9	61.7	40.9
≥4	36.8	38.1	38.3	39.8

*Women's decision-making power*				
Low	36.1	35.8	35.7	33.1
Moderate	33.4	33.0	32.3	35.9
High	30.5	31.2	32.0	31.0

**Table 3:** Unmatched logit model and propensity score matching of health insurance coverage associated with ANC, facility-based delivery, and postnatal care.

Wealth quintile	Maternal health care	Unmatched logit model				Propensity score-matched logit model			
		Unadjusted OR	95% CI	Adjusted OR	95% CI	ATEP *β*	95% CI	ATET *β*	95% CI
Poorest	ANC (≥4)	0.88	0.72, 1.08	1.03	0.81, 1.31	−0.02	−0.08, 0.05	−0.01	−0.08, 0.07
	Facility-based delivery	**0.79**	**0.63, 0.99** ^*∗*^	1.04	0.78, 1.39	−0.01	−0.07, 0.04	−0.01	−0.08, 0.05
	Postnatal care	0.91	0.74, 1.12	1.02	0.80, 1.30	−0.02	−0.08, 0.04	−0.01	−0.09, 0.07

Poorer	ANC (≥4)	0.98	0.72, 1.33	1.13	0.78, 1.62	0.05	−0.05, 0.07	−0.05	−0.12, 0.03
	Facility-based delivery	1.30	0.79, 2.13	1.59	0.87, 2.89	0.01	−0.03, 0.05	0.00	−0.04, 0.05
	Postnatal care	1.05	0.79, 1.40	1.18	0.83, 1.67	−0.01	−0.08, 0.08	−0.02	−0.14, 0.09

Middle	ANC (≥4)	0.86	0.58, 1.27	0.95	0.59, 1.53	−0.02	−0.10, 0.06	0.01	−0.09, 0.11
	Facility-based delivery	0.59	0.28, 1.22	0.60	0.23, 1.60	−0.01	−0.05, 0.03	−0.01	−0.07, 0.06
	Postnatal care	0.74	0.50, 1.08	0.85	0.55, 1.33	−0.01	−0.09, 0.08	0.02	−0.10, 0.13

Richer	ANC (≥4)	1.39	0.76, 2.54	1.30	0.61, 2.75	0.03	−0.02, 0.08	−0.03	−0.10, 0.05
	Facility-based delivery	0.99	0.40, 2.47	1.10	0.35, 3.46	0.02	−0.01, 0.05	−0.00	−0.05, 0.05
	Postnatal care	0.87	0.55, 1.36	0.82	0.47, 1.43	0.02	−0.09, 0.13	−0.04	−0.13, 0.05

Richest	ANC (≥4)	**2.48**	**1.06, 5.83** ^*∗*^	2.58	0.90, 7.43	0.05	−0.00, 0.11	0.07	−0.00, 0.14
	Facility-based delivery	1.76	0.51, 6.12	4.12	0.49, 34.39	**0.05**	**0.01, 0.10** ^*∗*^	0.07	−0.01, 0.15
	Postnatal care	1.59	0.93, 2.74	1.86	0.94, 3.65	**0.11**	**0.01, 0.20** ^*∗*^	**0.18**	**0.07, 0.29** ^*∗*^

OR = odds ratio; *β* = regression parameter; CI = confidence interval; ^*∗*^significant at *p* < 0.05; ATEP = average treatment effect in population; ATET = average treatment effect on the treated, model adjusted for age, place of residence, region, religion, reading newspaper/magazine, listening to radio, watching TV, maternal education, child wanted, age at first birth, and parity.

**Table 4:** Concentration index (CI) of ANC, facility-based delivery, and postnatal care by household wealth quintile and health insurance, Gabon DHS, 2012.

Factor	ANC			Facility-based delivery			Postnatal care		
	Health insurance	No health insurance	Overall	Health insurance	No health insurance	Overall	Health insurance	No health insurance	Overall
Conc. Index	0.125	0.096	0.117	0.076	0.053	0.069	0.078	0.063	0.075
SE	0.007	0.008	0.005	0.005	0.005	0.003	0.008	0.008	0.006
*P* value^*α*^	<0.001^*∗*^	<0.001^*∗*^	<0.001^*∗*^	<0.001^*∗*^	<0.001^*∗*^	<0.001^*∗*^	<0.001^*∗*^	<0.001^*∗*^	<0.001^*∗*^
*Health insurance coverage comparisons*									
z-stat	2.69			3.38			1.26		
Conc. Index difference	0.029			0.023			0.015		
*P* value^*β*^	0.007^*∗*^			0.001^*∗*^			0.209		

Conc. Index = Concentration Index; SE = standard error; ANC = antenatal care; ^*∗*^Significant at *p* < 0.05.

## Data Availability

Data for this study were sourced from Demographic and Health surveys (DHSs) and are available at http://dhsprogram.com/data/available-datasets.cfm.

## Abbreviations

UHC: Universal Health Coverage
SDGs: Sustainable Development Goals
NFHISG: National Fund for Health Insurance and Social Guarantee of Gabon
WHO: World Health Organization
SSC: Social solidarity contribution
EMCCA: Economic and Monetary Community of Central Africa
ANC: Antenatal care.
